# Learning multi-agent cooperation

**DOI:** 10.3389/fnbot.2022.932671

**Published:** 2022-10-14

**Authors:** Corban Rivera, Edward Staley, Ashley Llorens

**Affiliations:** ^1^Johns Hopkins Applied Physics Lab, Intelligent Systems Center, Laurel, MD, United States; ^2^Microsoft Research, Microsoft, Redmond, WA, United States

**Keywords:** multi-agent, policy learning, reinforcement learning, artificial intelligence, learned cooperation

## Abstract

Advances in reinforcement learning (RL) have resulted in recent breakthroughs in the application of artificial intelligence (AI) across many different domains. An emerging landscape of development environments is making powerful RL techniques more accessible for a growing community of researchers. However, most existing frameworks do not directly address the problem of learning in complex operating environments, such as dense urban settings or defense-related scenarios, that incorporate distributed, heterogeneous teams of agents. To help enable AI research for this important class of applications, we introduce the AI Arena: a scalable framework with flexible abstractions for associating agents with policies and policies with learning algorithms. Our results highlight the strengths of our approach, illustrate the importance of curriculum design, and measure the impact of multi-agent learning paradigms on the emergence of cooperation.

## 1. Introduction

Reinforcement learning (RL) offers a powerful approach to generating complex behaviors for intelligent systems that could not be explicitly derived or programmed. In the RL setting, the problem of learning an effective control policy is posed as a sequential decision-making problem for an agent interacting with a learning environment (Sutton and Barto, 2018). Given that learning the environment dynamics is an essential aspect of the RL problem, the ultimate effectiveness of a learned policy is dependent on the extent to which the learning environment reflects the essential aspects of the intended operating environment for the target system. Hence, many RL breakthroughs to date have focused on gaming and other applications with structured and predictable environments (Silver et al., 2016; Brown and Sandholm, 2019; Vinyals et al., 2019).

Translating progress in RL to increasingly complex applications of artificial intelligence (AI) will require the design of representative learning environments with corresponding complexity. Ensuring that future progress is reproducible and accessible for a broad community of researchers will require tools and frameworks that enable RL solutions to gracefully scale to address the problem of learning effectively in these increasingly complex settings. In general, RL frameworks must balance multiple tradeoffs, including ease of prototyping vs. training at scale, high-level abstractions vs. fine-grained control, and richness of features vs. ease of use.

## 2. Related work

Advances in the field of reinforcement learning has resulted in astonishing progress in the areas of robotic control (Lillicrap et al., 2015; Levine et al., 2016), and the ability to master challenging games (Mnih et al., 2015; Silver et al., 2016). To facilitate advancement in the field, numerous reinforcement learning frameworks have been developed to address scalable training (Caspi et al., 2017; Guadarrama et al., 2018; Schaarschmidt et al., 2018), reproducibility (Loon et al., 2019), robotics interoperability (Fan et al., 2018), lifelong learning (Fendley et al., 2022), ease of prototyping (Abel, 2019; Stooke and Abbeel, 2019; D'Eramo et al., 2020). Despite these advancements, these works are designed for the single-agent setting.

Our aim is to help enable RL research for the class of applications that involve multiple teams of agents where each team may have unique learning strategies and where agents within a given team may have localized views of the environment. Distributed multi-agent applications may be thought of as analogous to “system of systems” applications from a systems engineering perspective where collections (teams) of goal-oriented systems (agents) collaborate to achieve shared objectives. These attributes may arise, for example, in smart city applications where automated traffic control systems interact with fleets of automated vehicles (Shalev-Shwartz et al., 2016) or in defense applications (Cai et al., 2013) where heterogeneous autonomous systems interact across time and space to achieve high-level mission objectives. Applications such as these often include cooperation or competition (Busoniu et al., 2008) among heterogeneous teams of agents as defining features.

Progress in the area of multi-agent reinforcement learning has been made through the development of novel algorithms (Lowe et al., 2017; Rashid et al., 2018; Son et al., 2019) and frameworks to support distributed training of multi-agent policies (Zheng et al., 2017; Juliani et al., 2018; Liang et al., 2018). While these multi-agent focused frameworks make significant contributions to the field, these frameworks are designed to train with a single learning algorithm. Reinforcement learning algorithms have unique strengths and properties that make them ideal for different scenarios. Some of these properties include sample efficiency (Mnih et al., 2013; Haarnoja et al., 2018), shared value functions (Lowe et al., 2017), intrinsic curiosity (Haarnoja et al., 2018). A single learning algorithm may not be ideal for training the policies of all agents in a complex environment. For example, in environments where agents operate at different timescales, the agent interacting with the environment at the slowest timescale will collect the least experience. In these cases, sample efficient learning algorithms may be needed (Mnih et al., 2013; Haarnoja et al., 2018). For agents that rapidly interact with the environment, on-policy algorithms may achieve a desired level of average reward more quickly (Schulman et al., 2017). In the following section, we describe how our contributions address this important limitation.

## 3. Introduction to the AI arena

**Figure 1:**
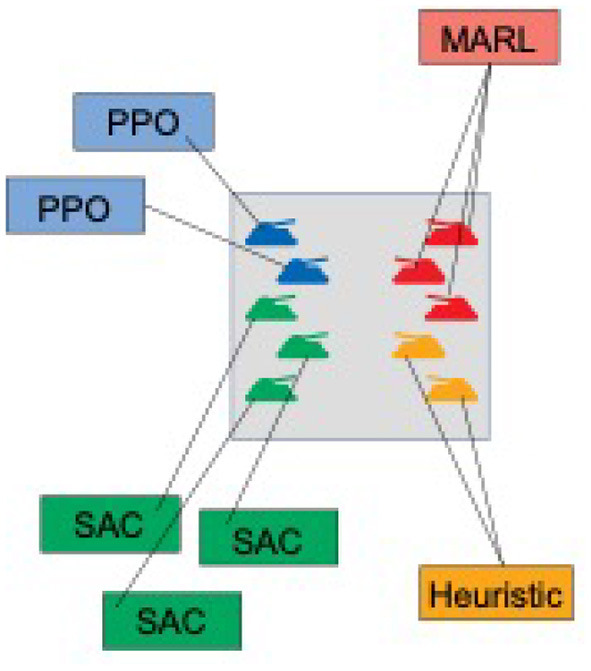
Simultaneous multi-agent multi-strategy policy learning. The figure illustrates an example multi-agent setting where the policies of individual agents are learned simultaneously with different learning strategies.

In this work, we introduce the AI Arena: a scalable framework with flexible abstractions for distributed multi-agent reinforcement learning. A key contribution of the AI Arena framework is the introduction of abstractions to flexibly associate agents with policies and policies with learning algorithms or heuristics. Figure 1 illustrates an example environment with flexible associations between agents, policies, and learning algorithms. The framework naturally distributes experience gathering over multiple nodes and routes those experiences to the associated learning algorithms to update policies.

### 3.1. Multiagent environments

One primary goal of the AI Arena interface is to encourage environments in which a variety of agents may coexist and learn together. This should encompass everything from collaboration among identical entities to competition among several dissimilar groups. To that end, other properties of the environment are also converted to lists, such as action spaces or observation spaces. This allows for a variety of agent types to coexist in a single environment. For example, one learning entity may be making discrete decisions about image state data, while another entity in the same environment may expect continuous actions based on a vectorized state space.

An implication of this multi-entity setup is that all entities, as well as their actions, observations, and rewards, are occurring in lock-step at the same rate. Each step, the environment expects decisions corresponding to all entities, and will return information to all of them about the consequences of those decisions. While this may seem limiting at first, it is better to think of this as supporting the most extreme case of multi-agent interaction: all entities can be involved in a single frame of the environment. It is fairly straightforward to embed special cases within this framework: an entity which has exited an episode early can send and receive null values, or an entity with a lower interaction frequency can easily on every *N*th observation and repeat actions until that observation occurs. The global “done” signal is especially useful for simulations and games in which there is a common or mutually exclusive objective, as is often the case.

### 3.2. Multiple learning policies

**Figure 2:**
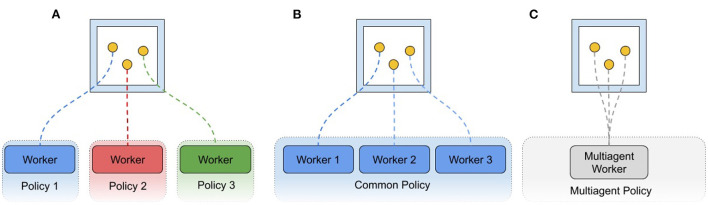
Possible worker configurations. Policy workers may be attached in environment entities in any desired combination. **(A)** Three entities are assigned to three independent policies that are learning separately and may have workers attached to other environments. **(B)** Entities are each attached to workers of the same policy, such that some of the agents contributing to the policy are coexisting in the same environment. **(C)** Entities are all attached to the same policy worker, which takes all of their data into account in a multiagent manner, possibly one of many workers for a distributed multiagent algorithm.

A further goal of the AI Arena interface is to enable complex distributed training architectures in which many policies may be training simultaneously in shared environments. The policies may be several instances of the same algorithm or be entirely separate approaches to learning. The inclusion of many entities in a single environment breaks from a typical training paradigm of one policy-worker thread corresponding to one agent in one environment. Rather, it is up to the user of this interface to distribute the many entities in an environment (or across many environments in the distributed case) to as many agents as desired. For example, an environment with *N* agents may function as *N* workers to a single distributed algorithm, or on the other extreme, single workers to *N* distinct policies. They may also be grouped such that *M* agents are in fact controlled by a single instance of a multi-agent policy (Figure 2). In other words, the agency of a given entity is at the discretion of the user. While this is a potentially powerful paradigm, it can be complex to implement. Additional details about interfaces, architecture, capabilities are described in Appendix.

## 4. Results and discussion

In this section, we highlight results that illustrate some of the key features of the AI Arena. Our experiments were designed to (i) test the importance of curriculum design on agent performance in the Tanksworld environment, and (ii) measure learned cooperation among several multi-agent paradigms in a cooperative navigation environment. For each experiment, we describe the environment, experiment design, and results.

### 4.1. The impact of curriculum design in the Tanksworld environment

A curriculum is anything that results in non-stationarity over the course of training (Bengio et al., 2009). A lot has been written about the challenges of non-stationarity in multi-agent environments (Gronauer and Diepold, 2022). In this experiment, we explored the potential benefits.

#### 4.1.1. Environment

**Figure 3:**
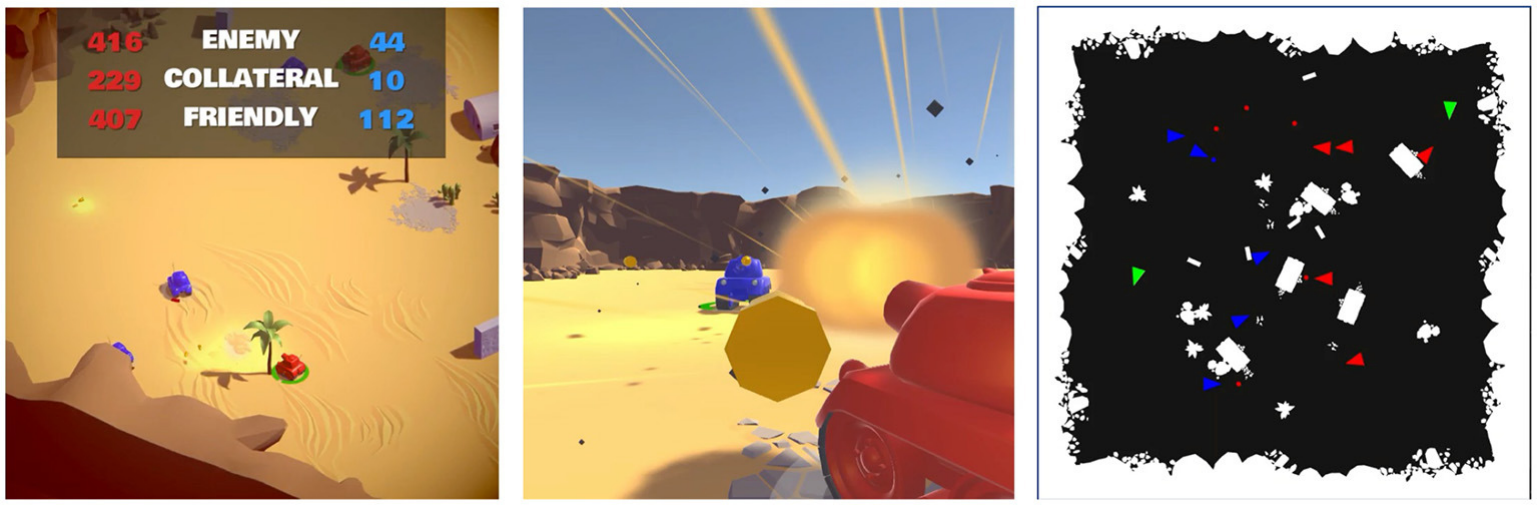
TanksWorld Multi-Agent Environment for AI Safety Research. These images illustrate different views of the Tanksworld environment **(Left)** is a birds-eye rendering of the environment, **(Center)** is an agent's-view rendering, and **(Right)** is the state representation actually provided as observations to the RL algorithm.

Illustrated in Figure 3, TanksWorld (Rivera et al., 2020) is a competitive 5 vs. 5 environment that challenges teams of agents to simultaneously win against the opposing team, cooperate with diverse teammates, and cope with uncertainty in the environment. The reward structure is a linear combination of rewards from enemy kills and damage and penalties for allied and collateral kills and damage. Additional details on the Tanksworld environment and reward structure can be found in the manuscript (Rivera et al., 2020).

#### 4.1.2. Experiment design

**Figure 4:**
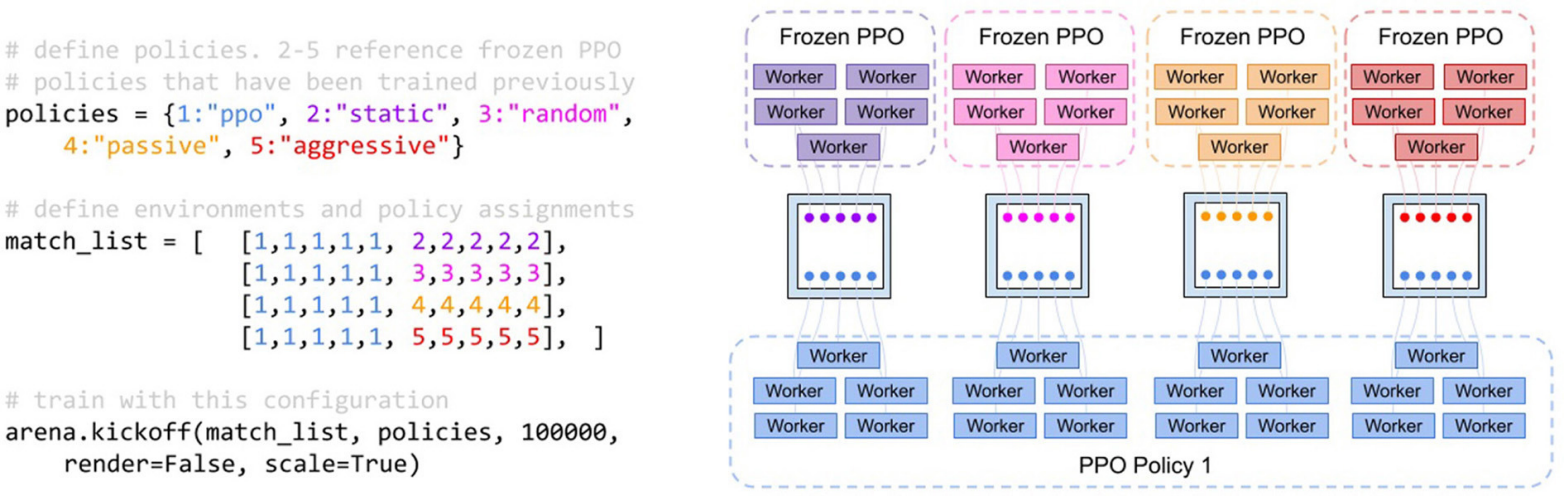
Pseudo-code and process diagram for the TanksWorld training scheme. The training scheme was used by both the static and dynamic curriculum. **(Left)** Pseudo-code of the training scheme against multiple policies. “ppo” refers to a PPO (Schulman et al., 2017) policy that is being trained, while the other policies refer to custom frozen policies. **(Right)** The resulting processes and their organization. Four environments are created, each housing 10 entities. The agents in the competition are depicted as nodes and colored based on the policies that they follow. All blue tanks are contributing to a single policy.

**Figure 5:**
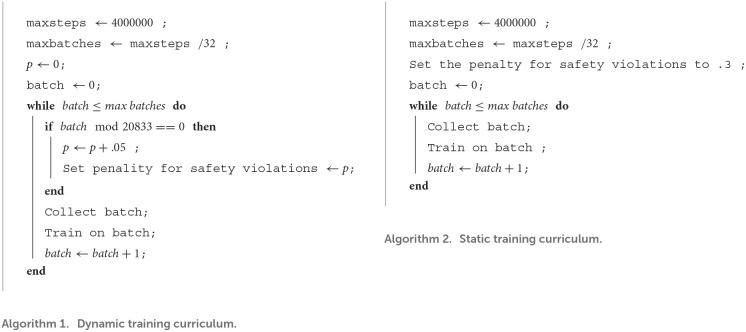
Pseudo-code for the dynamic vs. static training curriculum. The dynamic curriculum increased penalties for safety violations from 0 to 0.3 in increments of 0.05 distributed evenly over four million steps. The static curriculum keeps the penalty of safety violations at 0.3 throughout training. The safety violation parameter sets the penalty for damage or death caused by an ally to another ally or neutral entity.

The experiment compares reinforcement learning training against multiple opponents simultaneously with and without curriculum training. As shown in Figure 4, we train a policy with PPO (Schulman et al., 2017) against four different opponent policies (i.e., static, random, aggressive, and passive policies). The training scheme also illustrates the expressiveness of the abstractions in the AI Arena for multi-agent training. The policy weights for the aggressive and passive policies were pretrained with to saturation *via* PPO and frozen. Curriculum training was used to slowly introducing penalties for safety violations. The curriculum was composed of increasing penalties for safety violations from 0 to .3 in increments of 0.05 distributed evenly over four million steps. We compared the curriculum training approach to a baseline approach without a curriculum that sets the penalty for safety violations at 0.3. The difference between the static and dynamic curriculum training is illustrated by the algorithms in Figure 5. This means that the final reward configuration for both the static and dynamic curriculum cases were the same. We recorded average episodic reward over the number of steps in the environment.

#### 4.1.3. Results

**Figure 6:**
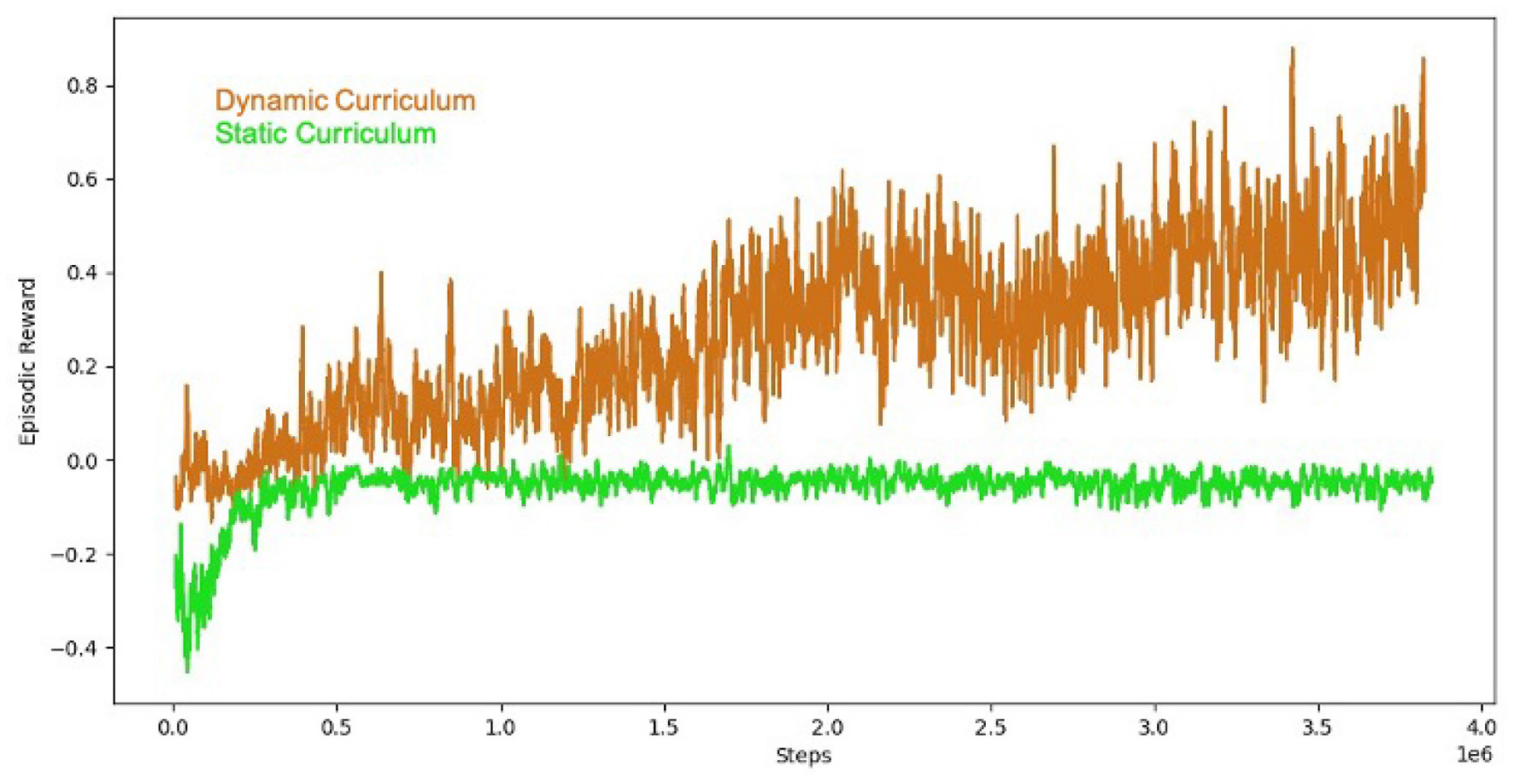
Round-based curriculum training organized with the AI Arena (brown). Successive rounds of training increased the difficulty by slowing introducing safety penalties over three rounds of training with penalty weights (0, 0.05, 0.1, 0.15, 0.20, 0.25, 0.3). The baseline (green) starts with the penalty threshold of 0.3. The result illustrates the value of successive rounds of curriculum training for teams of tanks in the AI Safety Challenge domain.

The results of the comparison are shown in Figure 6. The non-curriculum baseline reaches at plateau at just below 0, while the curriculum-based approach achieves a higher overall combined episodic reward which is a combined metric including both safety and performance. The early penalties for safety violations in the static curriculum inhibited exploration leading to a sub-optimal policy.

### 4.2. Learned cooperation with multi-agent soft actor critic

In this experiment, we aimed to better understand the effect of different multi-agent training paradigms using soft actor critic (SAC) (Haarnoja et al., 2018) on the emergence of cooperation. We evaluated cooperation using the cooperative navigation environment from MADDPG (Lowe et al., 2017). In the next section, we describe the environment in more detail.

#### 4.2.1. Environment

**Figure 7:**
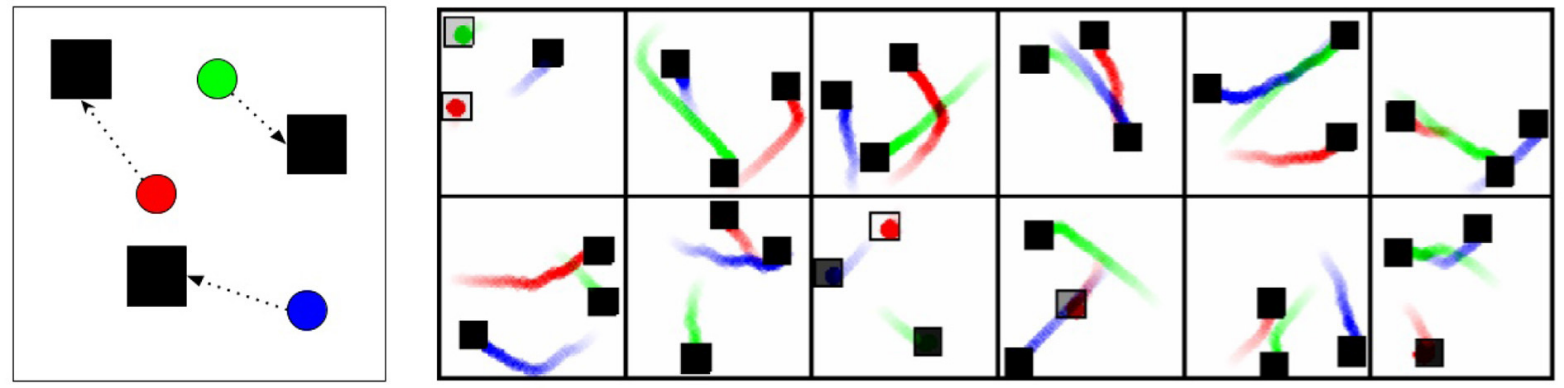
Cooperative navigation environment and behavior. The Cooperative Navigation environment has three targets (black squares) and three agents (circles). The agents must coordinate to cover all targets. **(Left)** Illustration of environment and potential solution. **(Right)** Traces of the testing behavior from the learned MASAC policies. The actions were reduced in magnitude to create slow paths to the targets. Some targets appear as outlines, showing that the agent happened to start on or near that target. The traces are interesting in that they show clear coordination but occasionally sub-optimal pairings of entities and targets. If the actions were not reduced, such that the entities reached the targets in only a handful of steps, these sub-optimalities would have little consequence on score.

The cooperative navigation environment is illustrated in Figure 7. In the environment there are 3 agents that can move in 2D space and must navigate to cover three targets. Targets provide a reward of +1 if they are occupied, so the optimal behavior is to have each entity travel to a unique target, such that all targets are occupied. There is no penalty for colliding with other agents. The environment runs for 300 steps, so the maximum theoretical score is 900 (all entities starting directly on a target and staying there for the duration, for 300 × 3 = 900 points).

#### 4.2.2. Experiment design

**Figure 8:**
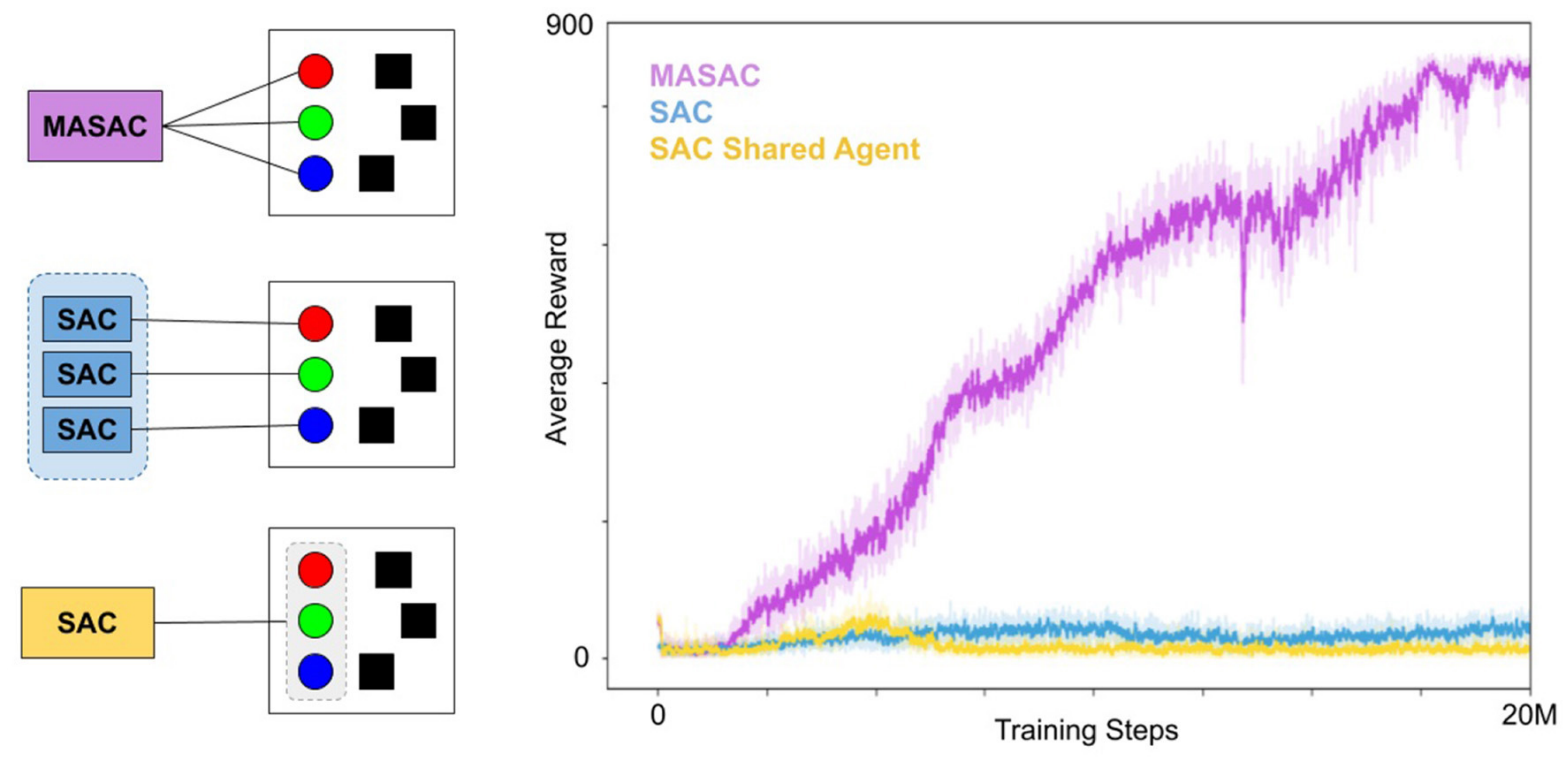
Training curves for MASAC and comparisons. **(Left)** Entity assignments for the three approaches: Truly multiagent policy (MASAC), treating each entity as an SAC worker, and grouping all entities into a single agent. In all cases, the assignment was duplicated over several environments for distributed training. **(Right)** The corresponding training curves for each approach. MASAC was the only successful algorithm, making slow and deliberate progress for roughly 18 million steps before leveling off.

We trained and compared three paradigms for multi-agent policy learning with SAC (Haarnoja et al., 2018) including (i) agents are controlled by individual SAC policies that share experience, (ii) a single SAC policy that controls all three agents, and a multi-agent variant of SAC (MASAC). These paradigms are illustrated in Figure 8 (left). Our implementation of Multi-agent Soft Actor Critic (MASAC) is a direct extension of soft actor critic (Haarnoja et al., 2018) to the multi-agent domain using the common critic framework initially described by MADDPG (Lowe et al., 2017). MASAC ran on eight environments (eight workers, each controlling three entities), SAC ran on six environments (18 workers, three per environment) and the combined entity SAC ran on eight environments (eight workers, each controlling three entities as one agent). All three approaches collected and trained using 20 M steps of experience.

#### 4.2.3. Results

As seen in Figure 8, our agents converge to a cooperative set of behaviors that clear 800 points on average, which is nearly optimal. The agents move quite quickly in this environment, but we have slowed them down in testing to create visualizations of their movements in Figure 7. While they do not always attempt to reach the nearest target, they have coordinated in such a way that all the targets become occupied. Crucially, they do not communicate during testing, so it is only through training with the common critic that they have learned complementary policies that can deploy independently while still working together.

The policy for individual control of converged at a low average reward as seen in Figure 8. Treating the agents as separate workers for SAC does not properly assign rewards to the agents, since all three agents are collectively rewarded based on target occupancy, and therefore the distributed SAC approach is not able to solve the credit-assignment problem among multiple workers. Similarly, the single SAC agent controlling all three entities converged at a low level of average reward. Treating all three entities as a single agent may suffer from a similar problem in that any rewards that are experienced do not reflect credit for the action taken but rather a subset of the action taken.

## 5. Conclusions

In this work, we introduced the AI Arena: a scalable framework with flexible abstractions for distributed multi-agent reinforcement learning. Our aim is to help enable RL research for the class of applications that involve multiple teams of agents where each team may have unique learning strategies and where agents within a given team may have localized views of the environment. Our experiment in the Tanksworld environment illustrated the importance of curriculum design, and our experiment with cooperative navigation highlighted the importance of multi-agent algorithms for the emergence of cooperative behavior.

## Data availability statement

The raw data supporting the conclusions of this article will be made available by the authors, without undue reservation.

## Author contributions

CR and ES jointly wrote the manuscript, produced the figures, ran the experiments, and developed the code. AL provided crucial guidance. All authors reviewed the manuscript. All authors contributed to the article and approved the submitted version.

## Conflict of interest

The authors declare that the research was conducted in the absence of any commercial or financial relationships that could be construed as a potential conflict of interest.

## Publisher's note

All claims expressed in this article are solely those of the authors and do not necessarily represent those of their affiliated organizations, or those of the publisher, the editors and the reviewers. Any product that may be evaluated in this article, or claim that may be made by its manufacturer, is not guaranteed or endorsed by the publisher.
