# Supplementary material for: Learning multi-agent cooperation
Source: Front Neurorobot. 2022 Oct 14;16:932671. doi: 10.3389/fnbot.2022.932671 (PMC9616006; doi:10.3389/fnbot.2022.932671)
Supplement: Supplementary file 1 [file Data_Sheet_1.pdf]

1 APPENDIX

1.1 AI ARENA INTERFACE

```python
# Basic AI Arena Interface:

def reset(self):
    # ...
    return list_of_initial_entity_states

def step(self, list_of_actions):
    # ...
    return list_of_states, list_of_rewards,
        done, list_of_info_dicts
```

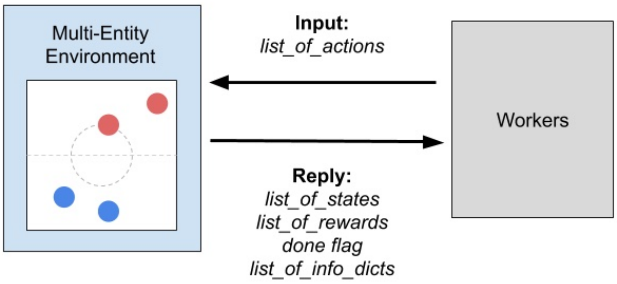

Figure 1. *The AI Arena Interface.* The AI Arena Interface is an adjustment to the OpenAI Gym interface such that many data objects are replaced with lists. This is easily added to an existing environment's reset() and step() methods such that a list of actions is accepted and lists of response data are returned.

The AI Arena Interface is an extension of the gym interface [2] that allows for environments to house multiple agents, without limitation on how those agents are attached to learning algorithms. This revision to the gym interface causes a distinct change in thinking about how environments are constructed: the environment is no longer completely representative of the problem a given agent is trying to solve. Rather, the environment is a shared space among one or more entities, each potentially having their own objectives. We use the term "entity" to avoid confusion between a policy and the portion of the environment under its control.

The above interface changes are manifested by simply expecting lists of values where the gym interface expects singular values. The only exception is the "done" flag, which is kept as a single boolean to indicate if the shared episode has ended. This data flow is described in Figure 1.

1.2 AI ARENA SOFTWARE FRAMEWORK

The AI Arena software framework is an implementation of the previously described AI Arena Interface with several goals in mind: 1) To allow usage of any environment or RL algorithm that the user may desire, with minimal effort, 2) To provide high-level functionality such that a user can quickly describe a very sophisticated training or testing scheme in python, and 3) to seamlessly orchestrate the desired training at scale.

1.3 PROCESS-BASED ARCHITECTURE

The AI Arena software framework is designed to run in a highly distributed and compartmentalized way, making heavy use of processes via the Message-Passing Interface (MPI) standard [3, 4]. Each environment instance is maintained in its own process, as well as each policy worker. The MPI paradigm allows many processes to spin up at once (across one or more machines), and allows various subsets of processes to communicate as illustrated in Figure 2. This is used by the AI Arena to designate messaging groups between an environment and any policy workers interfacing with that environment, as well as between collections of worker processes which all contribute to a single policy. These groupings are described in Figure 3, and details on environment and algorithm integration in the following subsections.

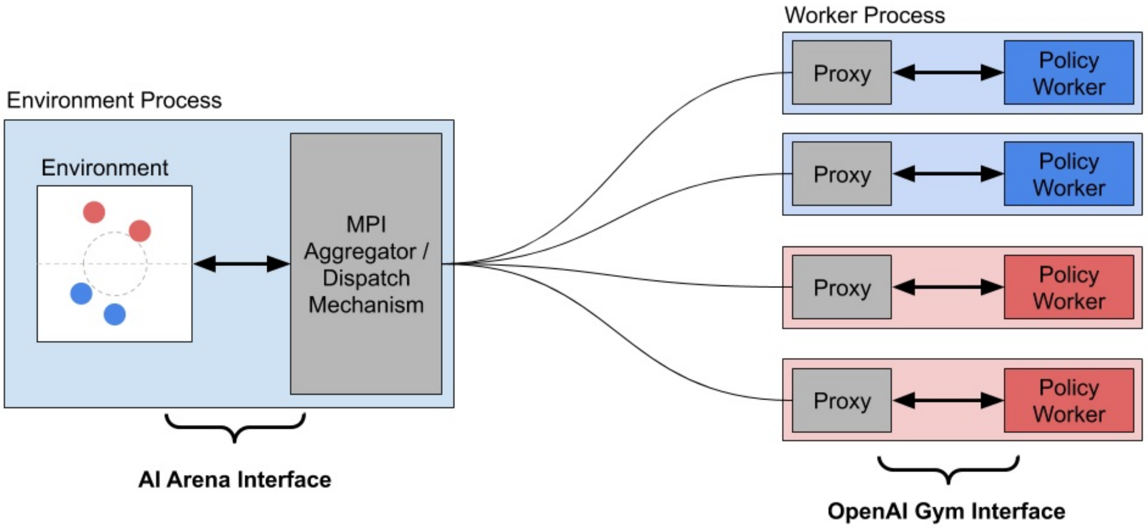

Figure 2. *Communication between an environment and single-agent policy workers.* **Left:** An environment instance is contained in a process which manages MPI communication. The core environment only needs to manage lists of actions and lists of replies. **Right:** Single-agent policy workers communicate locally with an environment proxy that handles MPI communication to a specific entity, expressed as a single-agent gym interface. Existing single-agent algorithms can be easily integrated using this paradigm.

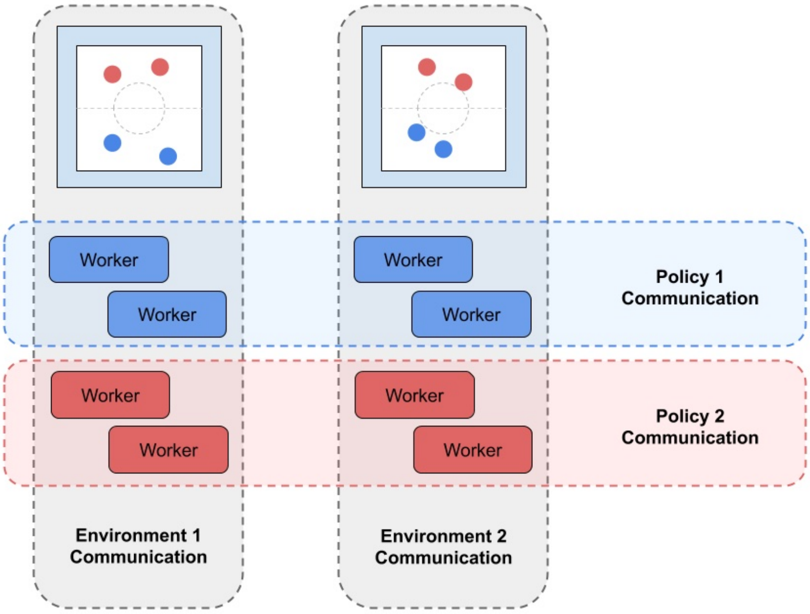

Figure 3. *Communication groupings among AI Arena processes.* MPI communication occurs between each environment and any policy workers which are commanding its entities. Separately, workers with a common policy communicate to share network weights, experience, or gradient information.

The AI Arena uses this grouping structure to orchestrate training runs with multiple environments, each having multiple entities, and contributing to multiple policies. This constitutes one training “rounds”.

While many reinforcement learning approaches train for an indeterminate amount of time, the AI Arena also handles the bringing down of distributed processes at specific times. This allows many rounds to be conducted seamlessly in sequence, enabling training schemes that progress through tournament structures, evolution of populations of agents, or schedules of environment parameters.

1.4 ENVIRONMENT INTEGRATION

As described previously, each environment is contained in a dedicated CPU process, and MPI is used to communicate between an environment and external agents. However, these details are fully obscured from a user looking to attach an environment. Each environment is wrapped in a python class which manages the communication to and from the environment, as well as MPI communication to and from policy workers. Actions from every relevant process are aggregated and sent to the worker as a single, local list communication, and replies are received as a list and then disseminated back to the respective parties (Figure 3 left). From the environment design perspective, the environment only needs to implement the previously described AI Arena Interface.

It is not uncommon for a python environment instance to itself manage an external environment, and the AI Arena places no restrictions on this design. For example, once an environment process is created by the AI Arena, an environment instance will be created. At this point, the python environment may bring up, for example, a Unity simulation which has its own lines of communication. This communication is completely separated from the AI Arena framework, just as the MPI calls are completely separated from the python environment instance.

1.5 ALGORITHM INTEGRATION

Just as the environment instances are contained in unique CPU processes, so are workers contributing to the training of a distributed reinforcement learning policy. MPI is again used here to maintain communication among a set of workers, to share gradients, data, or network parameters. Distributed algorithms looking to train with the AI Arena framework are encouraged to be set up in this manner, using MPI for updates as in [1]. However, other forms of communication could be used among processes at the user's discretion.

While the AI Arena Interface encourages highly multi-entity environments, many DRL algorithms are constructed to be single agent, and to communicate with the standard gym interface. To bridge this gap, the AI Arena software framework provides to each worker a proxy environment which implements the typical single-agent OpenAI gym interface, and communicates with the true environment on the backend. In this manner, a single-agent policy can be run without any modification. From its perspective, it is operating a single agent in a single environment, and may be unaware that other entities or other policies exist alongside it (Figure 3 right).

While this provides simple integration for many problems, it is not always sufficient to apply a single-agent algorithm to a multi-agent environment, and multi-agent algorithms have been developed for that use case. The AI Arena fully supports the assignment of multiple entities to a single policy worker rather than one, and in this case the worker will receive lists of data about several entities and be expected to reply with lists of actions. This enables, for example, a single worker process to manage all of the entities within an environment (or some subset). Scaling up to many environments, this would simply result in distributed training of a multi-agent algorithm. Furthermore, it is perfectly compatible to mix multi-entity and single-entity assignments, such that some entities may be attached to single-agent algorithms while others are pooled into multiagent algorithms.

```
# define policies
policies = {1:"ppo",2:"ppo",3:"ppo",4:"ppo"}

# define environments and policy assignments
# each match has slots for 4 policies
match_list = [
    [1,1, 2,2], [1,1, 3,3], [1,1, 4,4],
    [2,2, 3,3], [2,2, 4,4],
    [3,3, 4,4]
]

# train with this configuration
arena.kickoff(match_list, policies, 1000000,
    render=True, scale=True)
```

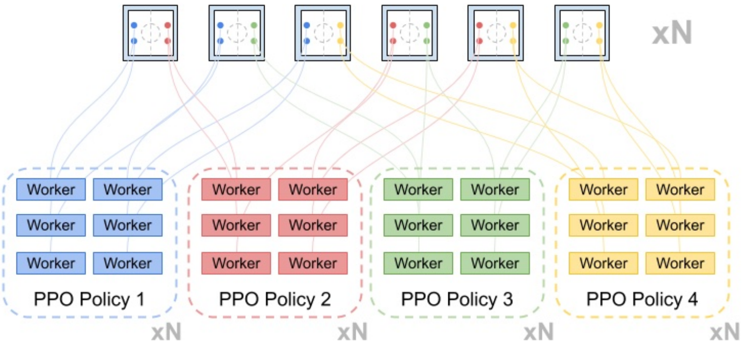

Figure 4. *Python code and process diagram for a four-policy round robin training scheme.* A hypothetical training scheme in which four policies train simultaneously in a round-robin style with a 2v2 environment. **Left:** Given an environment that conforms to the AI Arena Interface, the core content of the script to run this training scheme is only three lines. Four policies are defined, arranged into six environments, and the Arena is asked to run the provided configuration. **Right:** The resulting processes and their basic organization. With scaling turned on, this brings up $30N$ processes to run the configuration N times over simultaneously.

```
# define policy
policies = {1:"masac"} # or sac

# define environments and policy assignments
# each match has slots for 3 entities, which
# are grouped together for a single worker
match_list = [
    [[1,1,1]] #extra brackets to group entities
    # sac would just use [1,1,1] for 3 workers
]

# train with this configuration
arena.kickoff(match_list, policies, int(20e6/8),
    render=False, scale=True)
```

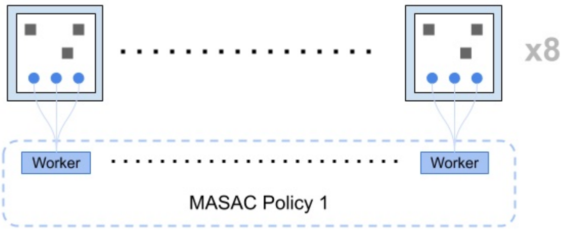

Figure 5. *Python code and process diagram for a multi-agent algorithm.* **Left:** Core code snippet needed to setup and run this training scheme. To route multiple entities to a single worker (as needed for a common critic algorithm), the entities are simple grouped by square brackets. Here we indicate that all entities 1-3 correspond to one worker for policy 1. **Right:** The resulting processes and their organization. In our experiments, eight environments were created, each having three entities but only one worker.

1.6 TRAINING DESCRIPTIONS AND SCALABILITY

Finally, the AI Arena software framework is designed to orchestrate much of the above implementation without any input from the user of the framework. Training schemes that previously would be quite cumbersome to maintain can be described in only a few lines of code (Figure 4 left). Furthermore, the AI Arena software framework includes many built-in utilities and conveniences, including example environments and common algorithms. Notably, a complex training scheme can be automatically duplicated across available compute. For example, the round-robin setup described in Figure 4 can be automatically repeated N times over such that $6N$ environments exist, connected to $24N$ workers representing 4 policies (Figure 4 right). Policy hooks allow for automatic logging, saving, and restoration to ease the maintenance of such complex setups. Additional utilities allow for policies to be duplicated so that they may be developed

over several rounds in population-based approaches. Figure 5 illustrates an example configuration for training multi-agent algorithms at scale.

REFERENCES

[1] Achiam, J. (2018). Spinning up in deep reinforcement learning.(2018). *URL https://spinningup. openai. com*

[2] Brockman, G., Cheung, V., Pettersson, L., Schneider, J., Schulman, J., Tang, J., et al. (2016). Openai gym. *arXiv preprint arXiv:1606.01540*

[3] Gabriel, E., Fagg, G. E., Bosilca, G., Angskun, T., Dongarra, J. J., Squyres, J. M., et al. (2004). Open mpi: Goals, concept, and design of a next generation mpi implementation. In *European Parallel Virtual Machine/Message Passing Interface Users' Group Meeting* (Springer), 97–104

[4] Tesser, F. (2016). Distributed message passing with mpi4py. In *Euroscipy 2016*
